# Occipital Hypometabolism on FDG PET/CT Scan in a Child with Hodgkin's Lymphoma

**DOI:** 10.1155/2016/5476108

**Published:** 2016-11-14

**Authors:** Inci Uslu Biner, Ebru Tatci, Ozlem Ozmen, Atila Gokcek, Haci Ahmet Demir, Nadide Basak Gulleroglu

**Affiliations:** ^1^Department of Nuclear Medicine, Ataturk Chest Diseases and Thoracic Surgery Training and Research Hospital, Ankara, Turkey; ^2^Department of Radiology, Ataturk Chest Diseases and Thoracic Surgery Training and Research Hospital, Ankara, Turkey; ^3^Department of Pediatrics, Memorial Ankara Hospital, Ankara, Turkey; ^4^Department of Radiology, Ankara Hematology Oncology Children's Training and Research Hospital, Ankara, Turkey

## Abstract

It is known that Fluorodeoxyglucose (FDG) Positron Emission/Computed Tomography (PET/CT) images may be helpful for evaluation of brain function in newborns. Here we described the fluorine-18 [18-F] FDG PET/CT imaging findings of encephalomalacia due to perinatal asphyxia in a child with refractory Hodgkin's Lymphoma (HL) who underwent PET/CT scan to stage the primary disease. Prominent hypometabolism was incidentally detected in the occipital regions bilaterally apart from the FDG uptakes in the malign lymphatic infiltrations. This case highlights the potential coexistence of a malignancy and a functional brain disorder.

## 1. Introduction

Encephalomalacia, also named as cerebral softening, is an area of focal brain damage that is most commonly seen in areas with insufficient blood supply. Cystic encephalomalacia is a softening of neurological tissue within a cystic cavity of the brain in various sizes and usually occurs prenatally during mid-fetal life secondary to a congenital infection (e.g., cytomegalovirus (CMV)) or occlusion of a major cerebral artery. It may also occur secondary to birth asphyxia [1–3].

Neonatal encephalopathy (NE) is a general term to define cerebral injury that can be caused by a variety of conditions. The most common cause of NE is hypoxia-ischemia, which is clinically named as hypoxic ischemic encephalopathy (HIE). It is a significant complication of neonatal-perinatal asphyxia that can cause some severe and permanent neuropsychological sequela or death [4]. HIE can occur secondary to several conditions including hypoglycemia [5, 6].

When the cause is an obstruction of blood flow, the temporal and parietal lobes are the most expected sites to be affected as cystic encephalomalacia [2, 3] while thinning of cerebral cortex predominantly in parietooccipital lobes is reported in several studies in hypoglycemic conditions [7].

In examining infants with suspected hypoxic ischemic brain injury, the main method is Magnetic Resonance Imaging (MRI). It is known that FDG PET/CT images may be helpful for evaluation of brain function in newborns. Here we described the metabolic imaging patterns of encephalomalacia due to perinatal asphyxia by MRI images in a child with refractory Hodgkin's Lymphoma (HL) who underwent PET/CT to stage the primary disease.

## 2. Case Report

**Figure 1:**
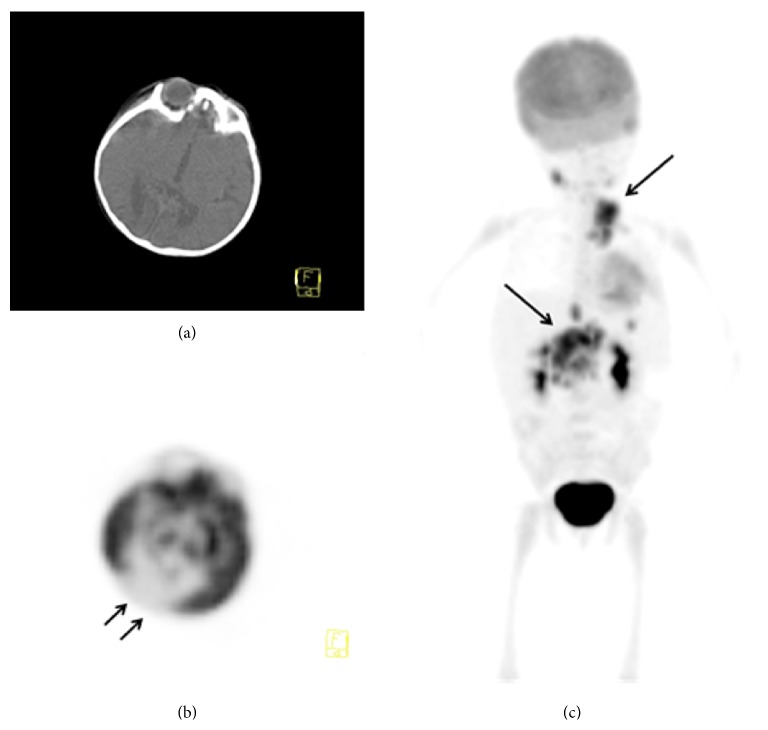
(a, b) In the cranial axial images of FDG PET/CT scan, hypometabolism in the occipital regions bilaterally was seen (arrows) and (c) maximum intensity projection (MIP) images of the staging PET/CT examination demonstrated focal increased FDG uptakes in the lymph nodes on the cervical and abdominal regions (long arrows).

**Figure 2:**
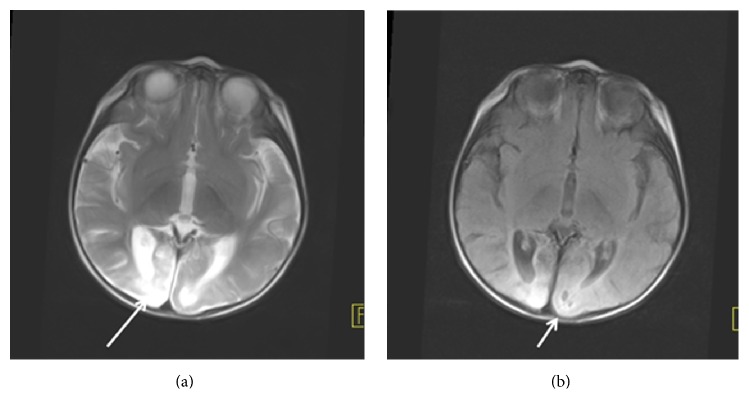
On brain Magnetic Resonance (MR) images, both visual cortices were hyperintense (a) (long arrow) on T2-weighted and flair weighted (b) (arrow) series.

A 3-year-old girl who was admitted to hospital with palpable masses in her neck and diagnosed as Hodgkin's Lymphoma (HL) was referred to our department for F-18 FDG PET/CT scan to stage the primary disease. She also had history of birth asphixia and congenital hypothyroidism with some clinical findings such as cerebral palsy, neurodevelopmental delay, seizures, and cortical blindness. She was suffering from seizures since she was three months old and started to take antiepileptics like Phenobarbital and Primidone. EEG was interpreted as showing epileptiform activity in the left temporoparietal region. Visual Evoked Potentials (VEP) showed abnormal visually evoked response (VER) with bilaterally affected nerves and deformed P1 waves. In the neurological examination, the deep tendon reflexes were normal if examined but mostly could not be examined since the child was agitated; the muscle tonus was increased in both upper and lower extremities without any pathological reflex. Additionally, she was microcephalic. She had not any seizure on the day of PET/CT scanning. She had not any history of brain operation and she and her parents had no history of any metabolic disorders and infections and that were retrospectively learned after evaluation of her PET/CT scan. Epstein Barr Virus sitology was negative. Also there is a history of consanguineous marriage of her parents. The glucose level was 40 mg/dL after birth but she did not have an episode of prominent and resistant hypoglycemia in the treatment period of lymphoma. The infant did not survive because of refractory HL. On cranial Computed Tomography (CT) images before the diagnosis of lymphoma, occupying mass lesion and hemorrhage were not seen in which volume loss and hypodensity were detected at the occipital region. FDG PET/CT images demonstrated prominent hypometabolism in the occipital regions bilaterally (Figures 1(a) and 1(b)) apart from the FDG uptakes in the malign lymphatic infiltrations in the cervical and abdominal regions (Figure 1(c)). On brain MRI, area of encephalomalacia was present in occipital region with surrounding gliosis. Both visual cortices were hyperintense on axial T2-weighted (a) series and in T2 and flair weighted series posterior parasagittal pattern of increased signal intensity in both occipital lobes (Figures 2(a) and 2(b)). On flair weighted series (Figure 2(b)), some subcortical cystic encephalomalacic changes in millimetric dimensions were seen and the thinning of the cerebral cortex also was noted in the occipital regions. MRI findings that were taken at different times (performed approximately interval of one year) were the same and not progressive. The diminished metabolic activity in PET/CT images was considered to be consistent with MRI findings and a part of encephalomalacia sequela. Informed consent was obtained from her parents prior to scanning.

## 3. Discussion

In our case, the findings of PET/CT and MRI were thought to be an encephalomalacia due to hypoglycemia and perinatal asphyxia because of the location and the corresponding clinical symptoms, the presence of the same MRI findings that were taken at different times. Neonatal encephalopathy may result from a variety of conditions. When caused by hypoxic ischemic brain injury, it is called HIE. HIE is one of the most common causes of cerebral palsy and other severe neurological deficits in children and either atrophy or white matter demyelination may be seen [4]. The most sensitive and specific imaging technique for examining infants with suspected hypoxic ischemic brain injury is MRI [8]. FDG PET/CT may provide information about metabolic status of brain tissue and may be a complementary to other methods in the estimation of probable neurologic sequela in patients with HIE due to perinatal asphyxia. Studies in the literature revealed that rates of glucose utilization in the areas of cortical scarring were lower than the other regions of the brain in term newborns with hypoxic ischemic encephalopathy [9]. Occipital hypometabolism was a constant finding in follow-up PET/CT images that were performed for the evaluation of therapy response of lymphoma in this case.

HL is uncommon in children under 10 years of age and it is rarely seen in children under 5 years [10]. Since the HL was not expected to occur at this age group of children, it was also interesting that this infant had a diagnose of HL at an earlier age than expected and the lymphoma was refractory; the infant did not survive because of the lymphoma.

Inflammation of the brain nearly always is associated with white matter lymphoma in the brain and leads to demyelination. Brain biopsy has an importance in the differential diagnosis of primary central nervous system lymphoma (PCNSL) and inflammatory demyelination while any suspected contrast-enhanced white matter lesion was examined [11]. In MRI images of our case, inflammation findings like meningeal thickening and suppression of the brain sulcus secondary to edema were not seen and white matter myelination was found to be behind her age. Unfortunately, there was not any MRI scanning of this child performed after the diagnosis of lymphoma because of her convulsions and clinical status and her parent's demand. Therefore, any demyelination that might be caused by white matter lymphoma could not be evaluated and we could not have a chance to compare the pre and post diagnose MRI findings. However, demyelination secondary to lymphoma was not considered with priority by taking into account the previous MRI findings.

Hypoglycemia is a rare complication of Hodgkin's disease [12]. However, MRI of this child was performed previously because of the convulsions and the scan findings mentioned above were detected before the diagnosis of lymphoma. Additionally, any hypoglycemic episode was not seen in this child during the diagnosis and treatment period of the lymphoma. Therefore, the correlated clinical and imaging findings of this child are thought to be more coincidental rather than a paraneoplastic hypoglycemia sequel.

In conclusion, this is a presentation of coexistence of HL and a functional brain disorder in a child. The coexistence of development of lymphoma at an earlier age and the presence of perinatal asphyxia history is challenging and it may be discussed to search the probable trigger effect of hypoxic condition to the development of a following malignancy in early infantile period with further studies. Also, this case reminds us of the importance of incidental findings that can be detected in PET/CT scans.
